# Factors influencing emergency dental care utilization among resettled Syrian refugee parents in Ontario: A cross-sectional study

**DOI:** 10.1371/journal.pgph.0004275

**Published:** 2025-02-12

**Authors:** Rhea Baba, Noara AlHusseini, Safoura Zangiabadi, Hala Tamim

**Affiliations:** 1 Department of Biology, School of Biomedical Sciences, Faculty of Science, McGill University, Montreal, Quebec, Canada,; 2 Department of Public Health, Alfaisal University, Riyadh, Saudi Arabia,; 3 Department of Kinesiology and Health Science, York University, Toronto, Ontario, Canada; Yale University School of Medicine, UNITED STATES OF AMERICA

## Abstract

Regular dental visits are crucial for maintaining optimal oral health, yet adequate access to oral healthcare services remains a significant challenge for refugee populations, including resettled Syrian refugees in Ontario. This study aimed to assess the factors associated with visiting the dentist only for emergency care among resettled Syrian refugee parents in Ontario. A cross-sectional study of 540 Syrian refugee parents, who had resided in Ontario for an average of four years and had at least one child less than 18 years of age, was conducted from March 2021 to March 2022. Information about visiting the dentist only for emergency reasons was gathered through the question, “How often do you usually visit the dentist?” Respondents who indicated that they visit the dentist only for emergency care were categorized as “yes.” Multivariable logistic regression was performed to examine the relationship between each of the sociodemographic-, migration-, and health-related factors with the primary outcome of visiting the dentist only for emergency. 73% of respondents reported visiting the dentist only for emergencies. Factors associated with emergency dental visits included dental insurance, number of children, and self-rated oral health. Individuals without dental insurance, higher number of children, and poorer self-rated oral health were more likely to seek emergency dental care. These findings highlight the barriers to regular dental visits among Syrian refugees in Ontario and underscore the need for more inclusive and accessible dental care services for such vulnerable population to enhance their oral health outcomes.

## Introduction

Regular dental visits are key for the maintenance of optimal oral health, given the integral role they play in dental care [1]. Per the Canadian Dental Association’s recommendation, visiting the dentist every 6 months is most beneficial for patients, and qualifies as adequate dental care [1,2]. While some benefits of regular oral health care include better mental and social health [3,4], the most notable outcome is prevention of oral disease [1]. Inadequate dental care and failure to prevent oral disease can increase the risk of adverse health consequences [5]. A clear example is seen through oral inflammation’s link to diabetes and cardiovascular disease, which can be exacerbated by bacterial-induced periodontal diseases [6,7]. By acknowledging the importance of dental care comes the recognition that it is unequally accessible across diverse populations in Canada and poses a significant burden for many [8]. As the Canadian dental system is predominantly privatized, it remains largely inaccessible to a significant portion of the population, notably those belonging to low-income brackets or lacking insurance [8,9]. While these may be barriers that many Canadians face, specific minority groups, such as refugees, disproportionately feel the burden of oral health disparities [8,10].

Canada hosts around thousands of refugees per year, including a significant number of Syrian refugees following the onset of the civil war in 2011 [10]. With 22 million individuals having been displaced since then, Canada has welcomed approximately 45,000 Syrian refugees between 2015 and 2023 [11]. As of December 31st 2023, the province of Ontario holds half of this population; around 19,635 Syrian refugees [11]. Studies indicate that dental care ranks high among refugees’ most immediate needs at arrival [12]. A recent study conducted among Syrian refugee parents in Ontario had participants self-rate their oral health, as well as provide information about their socioeconomic status, migration status, health, mental and dental health [13]. On a scale of very poor to excellent, 43.5% of participants self-reported their oral health as poor or very poor [13]. This is significantly higher than Canadians’ self-rated oral health of 5%. [14]. This percentage also exceeds that of a similar study surveying an Indigenous population in Canada, where 18.5% perceived their oral health as poor [15]. Clearly, Syrian refugees have the lowest levels of self-rated oral health among various Canadian populations, including other minority groups. The study conducted among Syrian refugee parents in Ontario showed several factors such as age, education and sponsorship to be associated with perception of their oral health. In addition, dental insurance was a significant predictor, with participants who had full or partial access to oral care insurance, exhibiting significantly improved self- rated oral health in comparison to those without insurance [13].

In Canada, provincial health coverage offered to refugees does not include oral health services [10]. Despite the efforts of making programs like the Interim Federal Health Program (IFHP) to offer dental care to refugees, the services are temporary, limited to emergencies or basic care, and open solely to those recognized by the Canadian government as refugees before their arrival [12].

Given the higher risk of poverty amongst refugees compared to other minority groups in Canada, private care is not a realistic option [12]. This leaves many refugees without essential dental care resources. Furthermore, Canada has shown a strong commitment to resettling Syrian refugees, with at least half of the resettled population residing in Ontario. Therefore, this study aimed to explore the factors associated with emergency-only dental care use among Syrian refugees in Ontario, Canada. Identifying the factors driving reliance on emergency dental services can offer valuable insights into unmet dental care needs in this underserved population and guide targeted interventions to improve dental service utilization and access, ultimately contributing to better overall health outcomes for Syrian refugees.

## Methods

### Study participants

This was a cross-sectional study of 540 Syrian refugee parents recruited and interviewed between March 3, 2021, and March 10, 2022. Participants eligible for inclusion needed to be Syrian refugee parents living in Ontario, Canada, who had at least one child under the age of 18 at the time of the interview and had resettled in Canada after 2015. Participants’ recruitment and interviews were conducted via telephone to adhere to COVID-19 social distancing guidelines. The recruitment process utilized convenience sampling facilitated by local service organizations, including Access Alliance Multicultural Health and Community Services and the Arab Community Centre of Toronto. The survey was administered by Research Assistants proficient in reading, writing, and speaking Arabic, particularly in the Syrian dialect.

### Ethical considerations

The study received ethical approval from the York University Research Ethics Board (Certificate # e2019-128) and was conducted in accordance with the ethical standards of the Helsinki Declaration. Before starting the survey, Research Assistants sent a digital copy of the consent form to the participants. Additionally, prior to their participation in the study, participants were informed about the study’s objective and the voluntary nature of their involvement, with the option to withdraw at any time without any consequences. Furthermore, individuals who chose not to participate received the same treatment and benefits as those who did. Research Assistants then thoroughly explained the consent form and addressed any questions or concerns from the participants. Following this, oral consent was obtained from the participants and documented for the record. To compensate for participants for their time and involvement, each individual received a $20 honorarium.

### Assessment of demographic characteristics, emergency-only dental visits, migration factors, and health-related factors

The main outcome of the study was visiting the dentist only for emergency care and was determined by the question “How often do you usually visit the dentist?”. For this question, respondents were provided with the following options: “more than once a year for check-ups”, “about once a year for check-ups”, “less than once a year for check-ups”, and “only for emergency care”. This variable was classified into a binary category of “yes” or “no”, with “yes” indicating visiting dentist only for emergency care. Moreover, other variables including sociodemographic-, migration-, and health-related factors were collected from the participants as potential predictors of visiting the dentist for emergency care. The sociodemographic characteristics considered included gender (being a mother/father), age (≤ 35/ 36- 45/ > 45), number of children (≤ 2/ 3-4/ >4), highest level of education (none-elementary/ secondary-high school-diploma/ university), employment status (yes/ no), self-perceived socioeconomic status (SES) measured by the question, “In your current condition, here in Canada, would you say most people would categorize a household like yours as” (Lower income/Lower middle income and middle income/Upper middle income and upper income), and dental insurance (yes/ no).

The migration-related factors comprised type of sponsorship (government-assisted refugee (GAR)/ privately sponsored refugee (PSR)/ and other), language proficiency in English or French (excellent-good/ fair-poor/ very poor-not at all), and years in Canada (≤4/ >4). Lastly, health-related factors encompassed self-rated mental and physical health (excellent/ very good- good/ fair-poor), smoking cigarettes (yes/ no), smoking narghile (yes/ no), alcohol (yes/ no), and self-rated oral health (excellent-good/ fair/ poor-very poor).

### Statistical analyses

Descriptive statistics of the main outcome and other variables were conducted. Simple logistic regression analysis was conducted to examine the bivariate association between all of the sociodemographic-migration-, and health-related factors with the main outcome of visiting the dentist only for emergency. Furthermore, multivariable logistic regression analysis was conducted after adjusting for all variables to predict the likelihood of visiting the dentist only for emergency care. Adjusted Odds Ratios (ORs), 95% CIs, and p-values were reported. Statistical significance was set to alpha = 0.05 for all analyses. All regression models were adjusted for the clustering effect of belonging to the same family. The analyses were conducted using the Statistical Package for the Social Sciences (SPSS, version 28.0).

## Results

Table 1 presents the descriptive statistics and the bivariate relationship between socio-demographic, migration, and health-related factors with visiting the dentist only for emergency care. Among participants, 72.8% reported visiting the dentist only for emergency care. Of the 540 participants, 60.9% were mothers, and 68.5% reported having more than two children. Additionally, slightly more than half of the participants (50.9%) were in the age range of 36-45. The majority (80.9%) had a secondary or university-level education, with 65.6% being unemployed. Furthermore, over half perceived their socioeconomic status as lower middle class or middle class, and 57.2% reported not having dental insurance.

**Table 1:** Results of the characteristics of study participants and the bivariate regression analyses of the association between sociodemographic-, migration-, and health-related factors and visiting dentist only for emergency care among Syrian refugee parents.

Factor	N	(%)	Unadjusted OR	95% CI	p-value
Socio-demographic					
**Gender**					
Mother	329	60.9	1		
Father	211	39.1	1.30	0.87, 1.95	0.203
**Age**					
≤ 35	153	28.3	1		
36- 45	275	50.9	1.81	1.15, 2.84	**0.010**
> 45	111	20.6	1.48	0.85, 2.58	0.169
**Number of Children**					
≤ 2	170	31.5	1		
3-4	264	48.9	1.20	0.78, 1.85	0.404
>4	106	19.6	3.81	1.90, 7.63	**<0.001**
**Education**					
None/ Elementary	101	18.7	2.87	1.49, 5.53	**0.002**
Secondary/High school/ Diploma/	278	51.5	1.42	0.92, 2.19	0.114
University/Professional	161	29.8	1		
**Employment**					
Yes	186	34.4	0.77	0.51, 1.15	0.195
No	354	65.6	1		
**Self-perceived SES**					
Lower	199	36.9	2.08	0.96, 4.51	0.064
Lower middle/ Middle	297	55.0	0.98	0.48, 2.03	0.966
Upper middle/ Upper	39	7.2	1		
**Dental Insurance**					
Yes	231	42.8	1		
No	309	57.2	4.52	2.95, 6.94	**<0.001**
Migration-related factors					
**Sponsorship**					
Governmental	202	37.4	1.54	1.01, 2.35	**0.047**
Private	312	57.8	1		
Other	26	4.8	2.33	0.76, 7.09	0.138
**Language Proficiency**					
Excellent/Good	195	36.1	1		
Fair/Poor	270	50.0	1.58	1.04, 2.38	**0.030**
Very poor/Not at all	75	13.9	2.48	1.25, 4.94	**0.010**
**Years in Canada**					
≤4	293	54.3	1.10	0.73, 1.65	0.646
>4	242	44.8	1		
Health-related factors					
**Self-rated physical health**					
Excellent	56	10.4	1		
Very good/ Good	342	63.3	1.75	0.96, 3.19	0.067
Fair/ Poor	142	26.3	2.25	1.14, 4.42	**0.019**
**Self-rated mental health**					
Excellent	64	11.9	1		
Very good/ Good	279	51.7	1.14	0.61, 2.12	0.687
Fair/ Poor	197	36.5	1.11	0.58, 2.13	0.742
**Smoking Cigarettes**					
Yes	119	22.0	1.60	0.97, 2.65	0.066
No	421	78.0	1		
**Smoking Narghile**					
Yes	138	25.6	0.75	0.49, 1.14	0.179
No	402	74.4	1		
**Alcohol**					
Yes	78	14.4	0.74	0.43, 1.26	0.270
No	462	85.6	1		
**Oral Health**					
Excellent/Good	179	33.1	1		
Fair	125	23.1	1.73	1.05, 2.86	**0.032**
Poor/Very poor	235	43.5	2.98	1.89, 4.71	**<0.001**

At the bivariate level, several socio-demographic, migration, and health-related factors were associated with visiting the dentist only for emergency care. Among socio-demographic factors, individuals with no or elementary level of education were almost three times at increased odds of visiting a dentist for emergency care than those with university-level education (OR=2.9, 95% CI: 1.49, 5.53). Also, those aged 36-45 and with more than four kids had a greater likelihood of emergency dental visits. Furthermore, not having dental insurance was linked with 4.5 increased odds of visiting the dentist for emergency care than those with dental insurance (OR= 4.52, 95% CI: 2.95, 6.94). Within migration-related factors, those with governmental insurance were at increased risk of seeking dental emergency care compared to those with private sponsorships (OR= 1.54, 95% CI: 1.01, 2.35). Comparably, those with poorer language proficiency in English and French were at increased odds of visiting the dentist for emergency care than those with excellent or good language proficiency. Amongst the health-related factors, having fair or poor self-rated physical health was associated with higher odds of visiting the dentist for emergency care.

Additionally, participants who rated their oral health as poor or very poor were at higher risk of visiting the dentist for emergency care than those with excellent self-rated oral health (OR= 2.98, 95% CI: 1.89, 4.71).

Table 2 indicates the results of the multivariable logistic regression. Not having dental insurance was significantly associated with visiting the dentist for emergency care. Participants without dental insurance were 5.8 times more likely to visit the dentist for emergency care than those with dental insurance (OR=5.80, 95% CI=3.54, 9.51). Also, number of children was significantly associated with visiting the dentist only for emergency care. Those participants with more than four children had an increased likelihood of visiting the dentist only for emergency care purposes than those with two children (OR=3.69, 95% CI=1.36, 10.03). Lastly, the results showed that those individuals who rated their oral health as poor or very poor had significantly higher odds of visiting the dentist only for emergency care than those with excellent or very good self-rated oral health (OR=2.21, 95% CI=1.28, 3.83, p=0.005).

**Table 2:** Results of multivariable logistic regression for the association between socio-demographic, migration, and health-related factors and visiting dentist only for emergency care among Syrian refugee parents.

Factor	Adjusted OR	95% CI	p-value
Socio-demographic			
**Gender**			
Mother	1		
Father	1.61	0.90, 2.89	0.111
**Age**			
≤ 35	1		
36- 45	1.37	0.79, 2.39	0.267
> 45	0.67	0.31, 1.46	0.314
**Number of Children**			
≤ 2	1		
3-4	0.88	0.52, 1.50	0.644
>4	3.69	1.36, 10.03	**0.011**
**Education**			
None/ Elementary	1.02	0.39, 2.67	0.962
Secondary/High school/ Diploma/	0.86	0.49, 1.50	0.597
University/Professional	1		
**Employment**			
Yes	0.83	0.47, 1.47	0.515
No	1		
**Self-perceived SES**			
Lower	2.13	0.83, 5.45	0.115
Lower middle/ Middle	1.08	0.46, 2.53	0.861
Upper middle/ Upper	1		
**Dental Insurance**			
Yes	1		
No	5.80	3.54, 9.51	**<0.001**
Migration-related factors			
**Sponsorship**			
Governmental	0.71	0.38, 1.31	0.269
Private	1		
Other	1.43	0.41, 5.01	0.576
**Language Proficiency**			
Excellent/Good	1		
Fair/Poor	1.23	0.73, 2.08	0.429
Very poor/Not at all	1.83	0.67, 5.04	0.240
**Years in Canada**			
≤4	1.02	0.63, 1.64	0.935
>4	1		
Health-related factors			
**Self-rated physical health**			
Excellent	1		
Very good/ Good	1.57	0.76, 3.27	0.224
Fair/ Poor	1.50	0.62, 3.60	0.370
**Self-rated mental health**			
Excellent	1		
Very good/ Good	0.93	0.43, 2.01	0.846
Fair/ Poor	0.59	0.25, 1.39	0.229
**Smoking Cigarettes**			
Yes	0.92	0.48, 1.75	0.796
No	1		
**Smoking Narghile**			
Yes	0.99	0.58, 1.67	0.961
No	1		
**Alcohol**			
Yes	0.79	0.40, 1.57	0.502
No	1		
**Oral Health**			
Excellent/Good	1		
Fair	1.53	0.86, 2.73	0.150
Poor/Very poor	2.21	1.28, 3.83	**0.005**

## Discussion

The present study examined the association between various socio-demographic-, migration-, and health-related factors with visiting the dentist only for emergency care among Syrian refugees in Ontario, Canada. Analysis revealed that 73% of participants visit the dentist solely for emergencies. The results of the study indicated that lack of dental insurance, having a greater number of children, and poorer self-rated oral health were significantly linked to increased likelihood of visiting the dentist only for emergency care. To effectively address the consequences of inadequate dental care among Syrian refugees, it is crucial to understand the range of factors influencing emergency dental visits. These insights underscore the urgent need for implementing new policies to ensure regular dental care for refugees.

The findings highlight that 73% of Syrian refugees resettled in Ontario visit the dentist only for emergency care. In contrast, immigrants in Ontario exhibit a lower rate, with 25% reporting visits to the dentist for emergencies only [16]. Similarly, other minority groups in Canada, such as the Indigenous population in Ontario, exhibit a comparable pattern of visiting the dentist, with 28% leave dental visits solely for emergencies [17]. However, these challenges are not exclusive to minority groups, as the predominant privatization of the Canadian dental system keeps access difficult for a large portion of the population [18]. A provincial survey conducted in Ontario reports that 19.3% of Ontarians visit the dentist only for emergencies [14]. While these studies may elucidate the disparities in emergency dental care utilization among various populations across Canada, it is evident that Syrian refugees are disproportionately impacted. A similar study conducted on Syrian refugees in Jordan demonstrates that 50% of the refugees did not visit a dental clinic in the past year, while the remaining 50% sought emergency dental treatment for concerns such as toothaches or dental infections [19].

The study revealed a significant association between the number of children a mother has and visiting the dentist only for emergency care. Mothers with more than four children exhibited a significantly higher likelihood of visiting the dentist only for emergency care compared to those with four or fewer children. This may be explained by the greater challenges families with a higher number of children face in managing routine dental care, which could lead to an increased frequency of emergency dental visits. Additionally, this finding may be attributed to mothers with more children often having lower incomes and lacking dental insurance, which can limit their access to regular dental care and increase reliance on emergency dental visits.[20]. Furthermore, working mothers are less likely to take their children to preventative dental appointments [21]. These factors underscore the challenges faced by parents with multiple children, leading to a higher use of emergency care instead of preventive care.

Another finding is that refugees without dental insurance were significantly more likely to visit the dentist only for emergency care than those with dental insurance. These findings align with previous studies, which indicate that individuals with low income or no insurance are four times more likely to avoid seeking dental care due to financial constraints [20,22,23]. Without dental insurance coverage, the fees can become excessively high, causing individuals to postpone treatment until symptoms worsen and emergency care becomes necessary. Despite government programs intended to help, these initiatives often have limitations that render them inaccessible for many refugees [12]. For instance, Canadian provincial health care for refugees does not cover dental visits [10]. These results highlight the critical role of insurance programs to alleviate the financial burden of dental care from Syrian refugees, enabling them to regularly visit the dentist.

An additional finding is that Syrian refugees with poor or very poor self-rated oral health are significantly more likely to seek emergency dental care than those with excellent self-rated oral health. This is consistent with a previous study, which shows a correlation between emergency-only dental visits and fair or poor self-rated oral health among immigrant populations in Ontario [16]. Some factors contributing to poor oral health perception include lack of insurance, governmental sponsorship, and irregular dental visits [13]. Another study suggests that reluctance to seek treatment and visiting the dentist for emergencies may be attributed to fear of discrimination, dental anxiety, or a perception of the importance of dental care [24].

Lastly, no association was found between smoking and seeking emergency dental care. This is contradictory to previous studies showing a general correlation between smoking and poor dental health [14,25,26]. For instance, smokers were 12.1% less likely to visit a dentist within the past year compared to non-smokers [25]. Possible reasons included avoiding treatment due to fear of judgment or financial constraints. Further investigation is needed to better understand the extent of the relationship between smoking and dental care.

### Limitations

While this study offers valuable insights into the association between socio-demographic, migration-related, and health-related factors and visiting the dentist only for emergency care, several limitations must be acknowledged. First, given the cross-sectional nature of this study, establishing the direction of causality between the variables may not be feasible. Second, there may be an information bias since all responses were self-reported. Third, there is a possibility of selection bias due to the voluntary nature of participation in the study. Fourth, information regarding dental health variables such as the count of decayed, missing, filled teeth, as well as flossing habits, was not collected. These omissions could potentially introduce confounding biases that influence outcomes related to emergency dental visits. Lastly, the use of the convenience sampling method limit the generalizability of the findings, as the sample may not represent the general population.

## Conclusion

This study examined patterns of dental care use solely for emergencies among Syrian refugees in Ontario and its associations to socio-demographic, migration-related and health-related factors. Analysis revealed that only 27% of Syrian refugees in Ontario reported visiting the dentist on a regular basis, compared to approximately 80% of Ontarians. Syrian refugees without dental insurance are at increased odds of visiting the dentist only for emergencies. These findings emphasize the need to focus efforts on improving dental care utilization among refugee populations, including Syrian refugees, to enhance their overall oral health. Establishing health policy reforms in provincial healthcare is essential to ensure that refugees, particularly those without insurance, have access to comprehensive dental care.
